# Cardiovascular risk and lipid-lowering therapy in idiopathic inflammatory myopathies: a call for improved, disease-specific risk assessment

**DOI:** 10.1093/rap/rkaf141

**Published:** 2025-12-06

**Authors:** Kimberly Loh, Emily Rose, Sarah Tansley

**Affiliations:** Rheumatology Department, Royal United Hospital, Bath, UK; Department of Life Sciences, University of Bath, Bath, UK; Rheumatology Department, Royal United Hospital, Bath, UK; Rheumatology Department, Royal United Hospital, Bath, UK; Department of Life Sciences, University of Bath, Bath, UK

Dear Editor, We read with interest the recent article by Llorca *et al.* evaluating the performance of cardiovascular (CV) risk scales in chronic inflammatory rheumatic diseases (CIRDs) [1]. The CARMA project provides important longitudinal data demonstrating the variability of different risk prediction tools in the estimation of CV events. Their findings underscore the challenges of accurately assessing and managing CV risk in this population. While the CARMA cohort included rheumatoid arthritis and spondyloarthritis, idiopathic inflammatory myopathies (IIMs) remain underrepresented in such validation studies. We would like to contribute by sharing data from our recent retrospective study examining CV risk estimation and lipid-lowering therapy use in patients with IIMs.

Evidence suggests that patients with IIMs are at increased risk of atherosclerotic CV mortality [2]. Optimising cholesterol and lipid management is one crucial component of CV health, yet statin use is underutilised, in part due to both patient and clinician concerns about statin-induced myopathy or muscle-related side effects [3]. While the available literature remains limited, existing evidence indicates that statins are generally well tolerated in non-anti-3-hydroxy-3-methylglutaryl-coenzyme A reductase (HMGCR) IIMs, and that alternative lipid-lowering agents (LLAs) may be safely employed in those with anti-HMGCR IIMs [4].

We performed a retrospective analysis of 74 patients attending the Myositis Clinic at the Royal National Hospital for Rheumatic Disease, Royal United Hospital, Bath, to evaluate cholesterol management and QRISK3 scores, with particular consideration of anti-HMGCR and non-anti-HMGCR subgroups. The work was done as a clinical audit to evaluate how well CV risk was being managed in IIM patients attending our clinic.

Of the 74 IIM patients, 10 were anti-HMGCR positive, 16 were seronegative and 48 had other myositis-specific antibodies. Ethnicity data were available for 71 patients: White-British, 52/71 (73.2%); White-other, 2/71 (2.8%); Asian 1/71, (1.4%); and Other-not stated, 16/71 (22.5%); 3 patients declined to answer.

Among the 10 anti-HMGCR IIM patients (median age 70.5 years, 40% female), 9 (90%) had previously received atorvastatin, with 3 also trialling an alternative statin. All presented with muscle symptoms and elevated creatine kinase, with levels ranging from 5000 to 19 393 IU/L. Following diagnosis, statins were switched to an alternative LLA in 6/9 (67%) and completely ceased in 3/9 (33%).

In the 64 non-anti-HMGCR IIM patients (median age 63.5 years, 73% female), 7 (11%) had previously used statins that were ceased with no alternative LLA prescribed. Seventeen (27%) remained on LLA therapy, 4 of which were switched from a statin to a non-statin LLA: 10 on atorvastatin, 3 on ezetimibe, 1 on cholestagel, 1 on ezetimibe/perbromic acid, 1 on rosuvastatin and 1 on rosuvastatin plus ezetimibe. Forty patients (63%) had never received lipid-lowering therapy.

QRISK3 scores were calculated for 56 patients within the valid age range (25–84 years) with available data. There was considerable variability, with scores of 17–36% in the anti-HMGCR group and 0.6–65.3% in the non-anti-HMGCR group. Median scores were similar between cohorts: 17.10% [interquartile range (IQR) 16.0–33.0] in HMGCR and 15.25% (IQR 7.0–24.4) in non-anti-HMGCR IIM, both considerably higher than that in the CARMA cohort. Alarmingly, despite moderate to high calculated CV risk (QRISK3 >10%), 14% of anti-HMGCR and 50% of non-anti-HMGCR IIM patients were not receiving any lipid-lowering therapy.

Traditional CV risk factors were more prevalent in the anti-HMGCR IIM cohort. Three (30%) had a previous cardiac event requiring intervention, 70% had hyperlipidaemia, 50% had diabetes, 10% had cerebrovascular disease and 50% had hypertension. The median BMI was 30.9 kg/m^2^ (excluding one missing value); 40% were former smokers and 60% were never-smokers. In comparison, the non-anti-HMGCR cohort had lower rates of comorbidities: 7.8% had prior cardiac events, 23.4% hyperlipidaemia, 10.9% diabetes, 4.7% cerebrovascular disease, 40.6% hypertension and 7.8% chronic kidney disease. Among 58 patients with available smoking data, 25.8% were former smokers, 6.9% current smokers and 67.2% never-smokers. The median BMI was 26.0 kg/m^2^ (excluding 13 missing values).

While the audit was designed to inform local practice, the findings provide general learning points relevant to the wider rheumatology community and align with the issues raised by Llorca *et al.* [1]. Our findings reveal that despite comparable QRISK3 scores, lipid-lowering therapy was underutilised in IIM patients, particularly in the non-anti-HMGCR subgroup. This reflects both physician and patient hesitancy in initiating statins, potentially due to concerns about muscle toxicity, as well as the need for improved risk assessment and education. Given the high prevalence of traditional CV risk factors and suboptimal lipid management, a proactive approach to cardiovascular prevention in IIM is warranted.

We commend Llorca *et al.* for highlighting the limitations of current CV risk calculators in CIRD. Our data similarly support the need for disease-specific risk prediction tools and greater awareness of lipid management in IIM. Future prospective studies should aim to validate CV risk prediction models specifically for myositis populations and evaluate safe, effective lipid-lowering strategies for both anti-HMGCR and non-anti-HMGCR subtypes.

## Data Availability

This study represents a retrospective clinical audit conducted at the Royal National Hospital for Rheumatic Disease, Royal United Hospital, Bath. De-identified data underlying this article may be provided upon reasonable request to the corresponding author.
